# The Dipeptidyl Peptidase-4 Inhibitor Teneligliptin Attenuates Hepatic Lipogenesis via AMPK Activation in Non-Alcoholic Fatty Liver Disease Model Mice

**DOI:** 10.3390/ijms161226156

**Published:** 2015-12-08

**Authors:** Takayasu Ideta, Yohei Shirakami, Tsuneyuki Miyazaki, Takahiro Kochi, Hiroyasu Sakai, Hisataka Moriwaki, Masahito Shimizu

**Affiliations:** 1 Department of Gastroenterology, Internal Medicine, Gifu University Graduate School of Medicine, 1-1 Yanagido, Gifu 501-1194, Japan; taka.mailbox.789@gmail.com (T.I.); tsunemiyazaking@yahoo.co.jp (T.M.); kottii924@yahoo.co.jp (T.K.); sakaih03@gifu-u.ac.jp (H.S.); hmori@gifu-u.ac.jp (H.M.); shimim-gif@umin.ac.jp (M.S.); 2 Informative Clinical Medicine, Gifu University Graduate School of Medicine, 1-1 Yanagido, Gifu 501-1194, Japan

**Keywords:** AMPK, DPP-4 inhibitor, lipogenesis, non-alcoholic fatty liver disease, NAFLD, SREBP1c, teneligliptin

## Abstract

Non-alcoholic fatty liver disease (NAFLD), which is strongly associated with metabolic syndrome, is increasingly a major cause of hepatic disorder. Dipeptidyl peptidase (DPP)-4 inhibitors, anti-diabetic agents, are expected to be effective for the treatment of NAFLD. In the present study, we established a novel NAFLD model mouse using monosodium glutamate (MSG) and a high-fat diet (HFD) and investigated the effects of a DPP-4 inhibitor, teneligliptin, on the progression of NAFLD. Male MSG/HFD-treated mice were divided into two groups, one of which received teneligliptin in drinking water. Administration of MSG and HFD caused mice to develop severe fatty changes in the liver, but teneligliptin treatment improved hepatic steatosis and inflammation, as evaluated by the NAFLD activity score. Serum alanine aminotransferase and intrahepatic triglyceride levels were significantly decreased in teneligliptin-treated mice (*p* < 0.05). Hepatic mRNA levels of the genes involved in *de novo* lipogenesis were significantly downregulated by teneligliptin (*p* < 0.05). Moreover, teneligliptin increased hepatic expression levels of phosphorylated AMP-activated protein kinase (AMPK) protein. These findings suggest that teneligliptin attenuates lipogenesis in the liver by activating AMPK and downregulating the expression of genes involved in lipogenesis. DPP-4 inhibitors may be effective for the treatment of NAFLD and may be able to prevent its progression to non-alcoholic steatohepatitis.

## 1. Introduction

Obesity is considered to be a serious health problem, as it frequently causes various medical concerns, including type 2 diabetes mellitus (T2DM), cardiovascular diseases, dyslipidemia and many types of cancer [1]. Non-alcoholic fatty liver disease (NAFLD), which is strongly associated with obesity, has become one of the most common causes of chronic liver disease in developed countries. The clinical importance of NAFLD is illustrated by its high prevalence (6.3%–33%, with a median of 20%) in the general population [2]. NAFLD is defined as a chronic hepatic status with fat accumulation in the liver after the exclusion of secondary causes of hepatic fat accumulation, such as remarkable alcohol consumption, autoimmune or viral hepatitis and certain medications [3]. Some patients with NAFLD develop a more serious disease condition, non-alcoholic steatohepatitis (NASH), and 10%–15% of patients with NASH develop liver cirrhosis, leading to hepatocellular carcinoma (HCC) [4,5,6]. The incidence of HCC due to NASH is almost the same as that due to chronic hepatitis C virus [7], which suggests that chronic liver damage or liver carcinogenesis associated with NAFLD/NASH are critical healthcare problems that should be resolved.

NAFLD is strongly associated with several aspects of metabolic syndrome, *i.e.*, obesity, dyslipidemia (primarily increased triglycerides), insulin resistance and concomitant glucose intolerance, including T2DM [6,8,9]. Therefore, improvement of these medical conditions may be beneficial to ameliorate NAFLD. For instance, pitavastatin, a drug used for the treatment of dyslipidemia, improved liver steatosis and decreased serum levels of free fatty acid (FFA) and alanine aminotransferase (ALT) in obese and diabetic *db*/*db* mice [10]. In the same strain of mice, treatment with green tea catechins, which have characteristics facilitating the prevention of metabolic syndrome, attenuated liver steatosis and suppressed chronic inflammation in the liver [11]. In addition, metformin, an anti-diabetic agent, markedly improve insulin resistance and inhibited obesity-related liver tumorigenesis in *db*/*db* mice [12]. Recently, it was reported that NAFLD is a strong determinant for the development of metabolic syndrome [13,14], suggesting that interventions purposing to ameliorate NAFLD are appropriate for the prevention and treatment of metabolic syndrome and related diseases.

Intestinal hormone incretins, such as glucagon-like peptide-1 (GLP1), regulate blood glucose levels by promoting insulin secretion in pancreatic β cells, as well as decreasing glucagon secretion in pancreatic α cells. Following their secretion from the intestines, incretins are rapidly decomposed by dipeptidyl peptidase (DPP)-4. DPP-4 inhibitors prevent GLP1 from decomposing, and this leads to appropriate secretion of insulin and glucagon from the pancreas. Therefore, DPP-4 inhibitors are commonly used in practice as medicinal agents for T2DM [15,16]. Recently, incretins have been reported to have various bioactivities, not only in pancreas cells, but also outside the pancreas [17]. Moreover, several studies have revealed the potential roles of incretin-based therapies, including DPP-4 inhibitors and GLP-1 receptor agonists, in the treatment of NAFLD [18,19]. DPP-4 inhibitors may be able to attenuate the pathology of NASH, because patients with NAFLD/NASH have increased DPP-4 activity, which correlates with the histological severity of NASH [20,21,22].

Monosodium glutamate (MSG)-treated animals exhibit obesity and metabolic dysfunction [23,24,25]. In the present study, we established a novel mouse model of NAFLD by injecting them with MSG and then feeding them a high-fat diet (HFD); these mice display obesity and severe fatty changes in the liver with an early onset. Using this model, we evaluated the preventive and therapeutic efficacy of teneligliptin, a DPP-4 inhibitor, on NAFLD and investigated the underlying mechanisms.

## 2. Results and Discussion

### 2.1. Results

#### 2.1.1. General Observations

At the end of the experiment, there were no significant differences in body weight or relative weight of organs, including the liver and white adipose tissue (periorchis and retroperitoneum), between the two groups (Table 1). No significant difference was seen in the amount of food ingested by the two groups during the experiment. No clinical symptoms of adverse event by teneligliptin were observed throughout the experiment. Histopathological examination also displayed no toxicity due to teneligliptin treatment in important organs, including the liver, kidney and spleen (data not shown).

**Table 1:** Body, liver and fat weights of the experimental mice.

Measurement Item	Control	Teneligliptin
Body weight (g)	83.4 ± 7.1 ^a^	80.7 ± 8.3
Liver weight (g)	5.5 ± 1.4	5.1 ± 0.8
Liver-to-body weight ratio	0.066 ± 0.013	0.063 ± 0.016
White adipose tissue ^b^ (g)	2.8 ± 0.7	2.8 ± 1.1

^a^ Mean ± SD; ^b^ white adipose tissue of the periorchis and retroperitoneum.

#### 2.1.2. Effects of Teneligliptin on the Histopathology of the Experimental Mouse Liver

**Figure 1:**
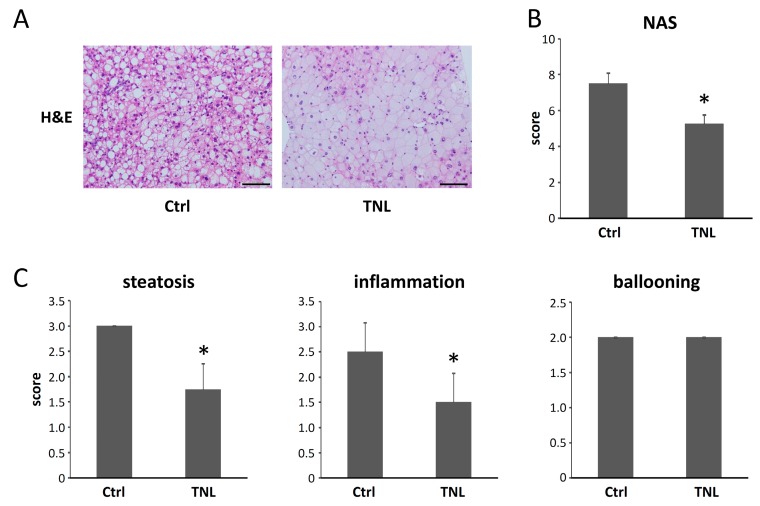
Effects of teneligliptin on hepatic histopathology in experimental mice. (**A**) Hematoxylin and eosin (H&E) staining of liver sections from experimental mice. Representative photomicrographs of the liver sections of MSG/high-fat diet (HFD)-administered mice treated with or without teneligliptin. Bar, 100 μm; (**B**,**C**) The NAFLD activity score (NAS) was determined based on histopathological analysis (steatosis, inflammation and ballooning). Ctrl, control. TNL, teneligliptin. The values are expressed as the mean ± SD. * *p* < 0.05 *versus* the control group.

The hematoxylin and eosin (H&E)-stained liver sections showed fatty degeneration, inflammation and hepatocellular ballooning in both groups. Macrovesicular fat deposits and glycogen storage were observed in the livers of both groups, but teneligliptin treatment attenuated fat accumulation in the experimental mice (Figure 1A). Liver sections were histologically evaluated using the NAFLD activity score (NAS) system [26]. The total NAS in Group 2 was significantly decreased compared to that in Group 1 (Figure 1B). When comparing each scoring factor in the NAS system, hepatic steatosis and inflammation were significantly attenuated in Group 2 compared to those in Group 1 at this experimental time point (14 weeks of age) (Figure 1C). Liver fibrosis was not detected in either group.

#### 2.1.3. Effects of Teneligliptin on the Intrahepatic Triglyceride Levels and the Activation of AMP-Activated Protein Kinase in the Livers of Experimental Mice

**Figure 2:**
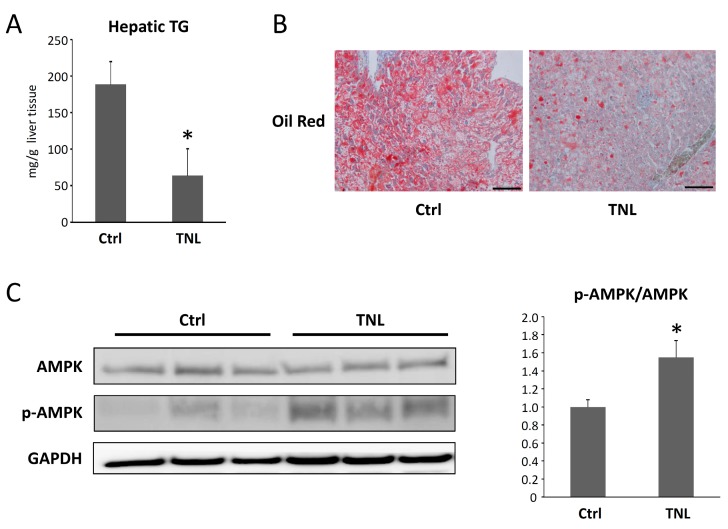
Effects of teneligliptin on hepatic steatosis and the levels of AMPK and p-AMPK in the livers of experimental mice. (**A**) Hepatic lipids were extracted from liver samples, and intrahepatic triglyceride (TG) levels were measured (*n* = 6); (**B**) steatosis in frozen liver sections from experimental mice treated with or without teneligliptin was analyzed with Oil Red O staining. Bar, 100 μm; (**C**) Total proteins were extracted from the livers of experimental mice, and the expression levels of AMPK and p-AMPK proteins were examined by Western blot analysis using the respective antibodies. GAPDH served as a loading control (**left** panel). Band intensities were quantified using densitometry. After the average of band intensity ratios of p-AMPK to GAPDH and AMPK to GAPDH were calculated in each sample, the ratios of these calculated values, which was expressed as p-AMPK/AMPK, were determined (**right** panel). Similar results were obtained in repeat experiments. The values are expressed as the mean ± SD. * *p* < 0.05 *versus* the control group.

Triglyceride levels in the liver were significantly decreased in the teneligliptin-treated group (Figure 2A). This was consistent with histological findings of attenuated hepatic steatosis in the livers of mice in the group treated with teneligliptin, as evaluated by Oil Red *O*-stained liver sections (Figure 2B). Moreover, teneligliptin administration significantly increased the hepatic expression levels of phosphorylated (*i.e.*, activated) AMPK (p-AMPK) protein (Figure 2C), which may be associated with the improvement of liver steatosis [27].

#### 2.1.4. Effects of Teneligliptin on the Expression Levels of Acetyl-CoA Carboxylase, Fatty Acid Synthetase, Sterol Regulatory Element-Binding Protein 1c and Elongation of Very Long Chain Fatty Acid-Like Family Member 6 mRNA in the Livers of Experimental Mice

**Figure 3:**
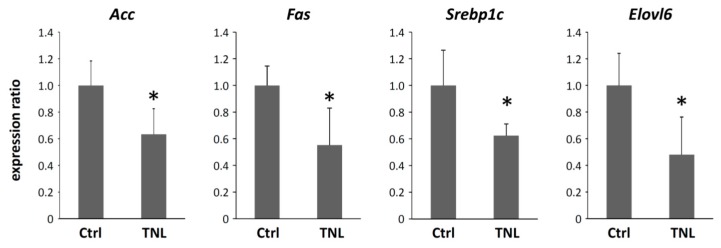
Effects of teneligliptin on the expression levels of genes related to lipogenesis in the livers of experimental mice. Total RNA was isolated from the livers of the experimental mice (*n* = 6), and the expression levels of *Acc*, *Fas*, *Srebp1c* and *Elovl6* mRNAs were examined using quantitative real-time RT-PCR with specific primers. The values are expressed as the mean ± SD. * *p* < 0.05 *versus* the control group.

We determined the mRNA expression levels of *Acc*, *Fas*, *Srebp1c* and *Elovl6* to elucidate the effects of teneligliptin on lipid metabolism in the livers of experimental mice. As shown in Figure 3, the expression levels of *Acc*, *Fas* and *Srebp1c*, which regulate lipogenesis [28,29], were significantly decreased in the mice treated with teneligliptin when compared to those without teneligliptin. In addition, teneligliptin administration also decreased the hepatic expression levels of *Elovl6*, which is also one of the key molecules controlling fatty acid metabolism and lipotoxicity [28].

#### 2.1.5. Effects of Teneligliptin on Biochemical Parameters

Blood samples were collected from the inferior vena cava at sacrifice after six hours of fasting for chemical analyses. The levels of serum ALT were significantly reduced by teneligliptin administration. On the other hand, other parameters, including FFA, glucose, insulin and triglyceride, were not significantly different between the groups (Table 2).

**Table 2:** Serum parameters in serum of the experimental mice. FFA, free fatty acid.

Measurement Item	Control	Teneligliptin
FFA (μEQ/mL)	2091.0 ± 328.9 ^a^	1550.4 ± 267.5
Glucose (mg/dL)	295.2 ± 108.2	528.0 ± 102.0
Insulin (ng/mL)	2.3 ± 0.9	2.14 ± 1.8
ALT (IU/L)	239.8 ± 20.4	162.0 ± 16.5 ^b^
Triglyceride (mg/mL)	56.4 ± 32.2	65.2 ± 9.3

^a^ Mean ± SD; ^b^ significantly different from the control group by the Welch *t*-test.

### 2.2. Discussion

The incidence of NAFLD/NASH is expected to continue to increase because of the global obesity epidemic. Therefore, efficacious therapeutic medications and preventive strategies for NAFLD/NASH are required. The novel animal model used in our present study is considered to reflect the pathological conditions in human NAFLD/NASH characterized by macrovesicular steatosis and chronic liver inflammation and is thought to be a practical and feasible model for investigating NAFLD and for testing preventive and therapeutic modalities that can suppress the progression of simple hepatic steatosis into NASH. In addition, this mouse model has the advantage of developing NAFLD with earlier onset compared to other animal models reported previously [11,23,30]. Although NAFLD/NASH has been considered as a hepatic manifestation of metabolic syndrome, it was recently found that NAFLD appears to be a precursor and a strong risk factor for the future development of metabolic syndrome [13,14]. A previous report by Misu *et al.* [31] suggested this reciprocal causality by demonstrating that the serum level of selenoprotein P, which is a liver-derived secretory protein and which is higher in subjects with NAFLD [32], causes insulin resistance. From this point of view, it is considered an appropriate action to intervene in ameliorating NAFLD by various medications, including the DPP-4 inhibitors, for the prevention and treatment of metabolic syndrome and related diseases.

DPP-4 inhibitors are commonly used in practice as medical agents for T2DM [15,16]. The present study clearly demonstrated that teneligliptin, a DPP-4 inhibitor, suppresses lipogenesis and steatosis in the liver of NAFLD model mice generated by administering MSG and HFD, whereas body weight and white adipose tissue weight were not reduced by this condition. We consider that the positive effect of teneligliptin on hepatic steatosis is associated, at least in part, with the suppression of the expression of specific genes, including *Srebp1c*, *Acc* and *Fas*, which play a key role in *de novo* lipogenesis [29]. *Srebp1c* is a key lipogenic transcription factor abundantly present in the mammalian liver [33]. It has been reported that hepatic gene expression of *Srebp1c* is increased in subjects with NAFLD as compared to those without [34]. In addition, treatment with linagliptin, the other DPP-4 inhibitor, also decreased liver expression of *Srebp1c* and *Fas* and, thus, improved steatosis in a mouse model of diet-induced obesity [35]. These reports may suggest that targeting lipogenic molecules, such as *Srebp1c* and *Fas*, with a DPP-4 inhibitor is a promising strategy for improving hepatic steatosis.

Among various agents investigated and thought to be candidates targeting NAFLD, the effects on fibrosis, ballooning degeneration, steatosis and lobular inflammation are analyzed in a recent publication comparing vitamin E, thiazolidinediones (TZDs), pentoxifylline and obeticholic acid (OCA) [36]. The effects of these agents are different; pentoxifylline, TZDs and OCA have ameliorating effects on lobular inflammation, but vitamin E has no effect on that compared to placebo. Furthermore, only pentoxifylline shows no effect on ballooning [36]. According to the results in our present study displaying the effects of teneligliptin on histopathology in the liver, teneligliptin could ameliorate hepatic steatosis and inflammation, but not ballooning in the NAS system (Figure 1). This might be because the major effect of teneligliptin as well as pentoxifylline [37] on NAFLD is inhibition of lipogenesis in the liver.

In the present study, the teneligliptin-treated group showed the tendency of a higher serum glucose level. This is assumed to be due possibly to the effect of fasting before sacrifice. In the feeding state, the serum glucose level must be lower than that in the control group, because the effect of this medicine on the serum glucose level has already been proven in experiments in the drug development process, as well as in clinical practice. Furthermore, in the feeding state, serum incretin levels appear to be higher in the teneligliptin-treated group, and it can be suspected that serum glucose metabolism was relatively dependent on the functions of incretins, including the functions that induce insulin secretion from the pancreas and enhance the insulin signaling pathway in the hepatocyte [17], due to the continuous influence of the DPP-4 inhibitor. Then, in the fasting state at sacrifice, intestines did not secrete incretins, leading probably to the relatively higher glucose levels shown in teneligliptin-treated mice. Although the serum levels of incretins and insulin, as well as glucose in the feeding state were not measured in our study, the levels of these might be able to let us interpret those unexpected data.

AMPK is a key regulator of energy balance and nutrient metabolism [38]. In the liver, AMPK has been demonstrated to inhibit cholesterol and triglyceride biosynthesis by reducing the activities of *Srebp1c* and *Fas* [27]. AMPK activation also promotes fatty acid β-oxidation by inactivation of ACC activity [39]. Moreover, GLP-1 suppresses hepatic lipogenesis through the activation of the AMPK pathway [40]. Other studies reported by Svegliati-Baroni *et al.* [41] and Lee *et al.* [42] also demonstrate that enhanced AMPK signaling due to GLP-1 activation can lead to inhibiting hepatic steatosis. Therefore, AMPK is considered to be a therapeutic target for NAFLD/NASH associated with metabolic syndrome [27]. In the present study, teneligliptin treatment significantly increased the levels of phosphorylated AMPK in the livers of NAFLD model mice (Figure 2C). These findings suggest that teneligliptin may attenuate lipogenesis in hepatocytes through the activation of AMPK and, subsequently, downregulation of *Srebp1c* and *Fas* (Figure 3). These findings are also consistent with the results of a previous report showing that AMPK inhibition resulted in elevated cleavage and transcription of hepatic *Srebp1c* in insulin-resistant mice [27]. In our study, it can be considered that teneligliptin elevated the level of GLP-1 due to attenuating the effect of the DDP-4 inhibitor and then enhanced AMPK in hepatocytes through the GLP-1 receptor (GLP-1R). The levels of GLP-1 and other incretins, however, were not determined in this study, as mentioned above. In addition, it is still controversial whether GLP-1R is present or responsible for the GLP-1 signal in the hepatocyte [43]. Moreover, there may be direct effects of DPP-4 inhibitors on hepatic steatosis through AMPK activation or other signaling pathways. Further investigations are required in order to clarify the effect of DPP-4 inhibitors and incretins on lipid metabolism in the hepatocyte.

One of the key mechanisms of incretin-based therapies, including DPP-4 inhibitors, for improving liver steatosis is the reduction of FFA [44] and improvement of glucose metabolism [15,16]. Therefore, we initially expected that teneligliptin would attenuate liver steatosis in the MSG/HFD-treated mice by improving these metabolic abnormalities. However, serum levels of FFA, glucose, insulin and triglycerides were not decreased by treatment with teneligliptin in the present study. We speculated that this was likely due to the study protocols, because MSG plus HFD treatment induced very severe obesity and steatosis within a short period of time. The present experimental condition (10 weeks of treatment with teneligliptin) may have been insufficient to obtain anti-diabetic effects, which is one of the limitations of the present study. Another limitation is that plasma levels of GLP-1 were not measured, and therefore, inhibition of DPP-4 by teneligliptin was not evaluated. We also did not assay the plasma DPP-4 activity or concentration. Therefore, future long-term studies should be conducted to confirm that teneligliptin improves liver steatosis by decreasing serum levels of FFA and improving glucose metabolism, focusing on the serum levels of GLP-1 and the activity of DPP-4 in several animal models.

## 3. Experimental Section

### 3.1. Animals and Chemicals

ICR mice were obtained from Charles River Japan (Kanagawa, Japan), and their newborns were employed in the study. MSG was purchased from Wako Pure Chemical (Osaka, Japan). CRF-1, a basal diet and HFD were from Oriental Yeast (Tokyo, Japan). Teneligliptin (Tenelia™) was kindly provided by Mitsubishi Tanabe Pharma Corporation (Tokyo, Japan). We fully complied with the Guidelines Concerning Experimental Animals issued by the Japanese Association for Laboratory Animal Science [45] and exercised due consideration to minimize pain and suffering.

### 3.2. Experimental Procedure

MSG was administered into the neonatal ICR mice at birth as a single-dose subcutaneous injection (4 mg/g body weight). Among these mice, males were divided into two groups at 4 weeks of age: the MSG/HFD group (*n* = 6, Group 1) and the MSG/HFD/teneligliptin-treated group (*n* = 6, Group 2). The mice in Group 2 were administered teneligliptin (30 mg/kg per day) in the drinking water from 4 weeks of age. The treatment dose of teneligliptin was determined according to the data from the animal experiments in the drug development process. Although the dose was relatively higher than that for humans in clinical practice, no notable adverse effect was observed in the treatment with the dose for the experimental animal in the process. Both groups were fed HFD from 4–14 weeks of age. At the termination of the experiment (14 weeks of age), all animals were sacrificed by CO_2_ asphyxiation to analyze hepatic histopathology.

### 3.3. Histopathological Examination

Maximum sagittal sections of three hepatic sublobes were used for histopathological examination. For all experimental mice, 4 μm-thick sections of formalin-fixed and paraffin-embedded livers were stained with H&E for conventional histopathology. The histological features of the liver were evaluated using the NAS system [26].

### 3.4. Clinical Chemistry

Blood samples were collected from the inferior vena cava at sacrifice after 6 h of fasting for chemical analyses. Unfortunately, one blood sample could not be taken properly in the sampling procedure in each group; therefore, 5 blood samples in each were used to analyze. The serum concentrations of glucose (BioVision Research Products, Mountain View, CA, USA), triglycerides (Wako Pure Chemical), FFAs (Wako Pure Chemical) and insulin (Shibayagi, Gunma, Japan) were measured as previously reported [46]. ALT was measured using a standard clinical automatic analyzer (Type 7180; Hitachi, Tokyo, Japan).

### 3.5. RNA Extraction and Quantitative Real-Time Reverse Transcription-PCR Analysis

Total RNA was extracted from the mice livers using the RNeasy Mini Kit (QIAGEN, Venlo, The Netherlands). cDNA was synthesized from 0.2 μg of total RNA with the High Capacity cDNA Reverse Transcription Kit (Applied Biosystems, Foster City, CA, USA). A quantitative real-time reverse transcription-PCR (RT-PCR) analysis was applied using a LightCycler Nano (Roche Diagnostics, Indianapolis, IN, USA) and FastStart Essential DNA Green Master (Roche Diagnostics). The sequences of specific primers for amplifying e*Elovl6*, *Fas*, *Acc*, *Srebp1c* and *18S* genes were obtained by Primer-BLAST [47] (Table 3). The expression level of each gene was normalized to that of *18S*.

**Table 3:** Primer sequences.

Genes	5′-Primer	3′-Primer
*Acc*	GGCTCAAACTGCAGGTATCC	TTGCCAATCCACTCGAAGA
*Elovl6*	CAGCAAAGCACCCGAACTA	AGGAGCACAGTGATGTGGTG
*Fas*	GCTGCTGTTGGAAGTCAGC	AGTGTTCGTTCCTCGGAGTG
*Srebp1c*	CTGGAGCTGCGTGGTTT	GCCTCATGTAGGAATACCCTCCTCATA
*18s*	CCATCCAATCGGTAGTAGCG	GTAACCCGTTGAACCCCATT

### 3.6. Hepatic Lipid Analysis

Approximately 200 mg of frozen liver samples were homogenized, and lipids were extracted using Folch’s method [48]. The triglyceride levels in the liver were measured with the Triglyceride E-test Kit (Wako Pure Chemical), as previously reported [49]. To visualize the intrahepatic lipids, Oil Red O staining was performed based on the standard protocol for frozen liver sections.

### 3.7. Protein Extraction and Western Blot Analysis

Total protein was extracted from the mice livers, and equivalent amounts of proteins (10 μg/lane) were examined by Western blot analysis [11]. Primary antibodies were obtained from Cell Signaling Technology (Beverly, MA, USA), including AMPK (#2603), p-AMPK (#2535) and GAPDH (#2118). The antibody for p-AMPK was used to detect the phosphorylation site at Thr172 in the activation loop. GAPDH served as the loading control. The intensities of the bands were quantified with NIH Image software ver. 1.62 (Bethesda, MD, USA). After the average of band intensity ratios of p-AMPK to GAPDH and AMPK to GAPDH was calculated in each sample, the ratio of these calculated values, which was expressed as p-AMPK/AMPK, were determined.

### 3.8. Statistical Analysis

The results are presented as the means ± SD and were analyzed using JMP software Version 10 (SAS Institute, Cary, NC, USA). Differences among the two groups were analyzed by Welch’s *t*-test. The differences were considered significant at *p*-values of less than 0.05.

## 4. Conclusions

Teneligliptin, the DPP4 inhibitor, improved the histopathological appearance of the liver and decreased intrahepatic triglyceride levels in an NAFLD model mouse, which was associated with downregulation of hepatic lipogenesis-related genes due to AMPK activation. Interestingly, the hepatic *Dpp-4* mRNA expression level is significantly higher in patients with NAFLD compared to healthy subjects [50]. The results of the present study, together with those of previous reports [19,21,22], have prompted us to conduct a clinical trial to determine the effectiveness of DPP-4 inhibitors for the prevention and treatment of NAFLD.
